# In vivo fluorescence imaging of the transport of charged chlorin*e6* conjugates in a rat orthotopic prostate tumour

**DOI:** 10.1038/sj.bjc.6690686

**Published:** 1999-09

**Authors:** M R Hamblin, M Rajadhyaksha, T Momma, N S Soukos, T Hasan

**Affiliations:** Wellman Laboratories of Photomedicine, WEL 224, Harvard Medical School, Massachusetts General Hospital, Boston, MA, 02114, USA

**Keywords:** photosensitizer, pharmacokinetics, photodynamic therapy, tumour vasculature, polymer conjugate

## Abstract

Polymeric drug conjugates are used in cancer therapy and, varying their molecular size and charge, will affect their in vivo transport and extravasation in tumours. Partitioning between tumour vasculature and tumour tissue will be of particular significance in the case of photosensitizer conjugates used in photodynamic therapy, where this partitioning can lead to different therapeutic effects. Poly-l-lysine chlorin*e6* conjugates (derived from polymers of average*M*_r_ 5000 and 25 000) were prepared both in a cationic state and by poly-succinylation in an anionic state. A fluorescence scanning laser microscope was used to follow the pharmacokinetics of these conjugates in vivo in an orthotopic rat prostate cancer model obtained with MatLyLu cells. Fluorescence was excited with the 454–528 nm group of lines of an argon laser and a 570 nm long pass filter used to isolate the emission. Results showed that the conjugates initially bound to the walls of the vasculature, before extravasating into the tissue, and eventually increasing in fluorescence. The anionic conjugates produced tissue fluorescence faster than the cationic ones, and surprisingly, the larger*M*_r_ conjugates produced tissue fluorescence faster than the smaller ones with the same charge. These results are consistent with differences in aggregation state between conjugates. © 1999 Cancer Research Campaign


*British Journal of Cancer* (1999) **81**(2), 261–268
© 1999 Cancer Research Campaign
Article no. bjoc.1999.0686

### In vivo fluorescence imaging of the transport of charged chlorin *e6* conjugates in a rat orthotopic prostate tumour

**MR Hamblin, M Rajadhyaksha, T Momma*, NS Soukos and T Hasan**

Wellman Laboratories of Photomedicine, WEL 224, Harvard Medical School, Massachusetts General Hospital, Boston, MA 02114, USA

**Summary** Polymeric drug conjugates are used in cancer therapy and, varying their molecular size and charge, will affect their in vivo transport and extravasation in tumours. Partitioning between tumour vasculature and tumour tissue will be of particular significance in the case of photosensitizer conjugates used in photodynamic therapy, where this partitioning can lead to different therapeutic effects. Poly-l-lysine chlorin *e6* conjugates (derived from polymers of average $M_r$ 5000 and 25 000) were prepared both in a cationic state and by poly-succinylation in an anionic state. A fluorescence scanning laser microscope was used to follow the pharmacokinetics of these conjugates in vivo in an orthotopic rat prostate cancer model obtained with MatLyLu cells. Fluorescence was excited with the 454–528 nm group of lines of an argon laser and a 570 nm long pass filter used to isolate the emission. Results showed that the conjugates initially bound to the walls of the vasculature, before extravasating into the tissue, and eventually increasing in fluorescence. The anionic conjugates produced tissue fluorescence faster than the cationic ones, and surprisingly, the larger $M_r$ conjugates produced tissue fluorescence faster than the smaller ones with the same charge. These results are consistent with differences in aggregation state between conjugates.

© 1999 Cancer Research Campaign

**Keywords:** photosensitizer; pharmacokinetics; photodynamic therapy; tumour vasculature; polymer conjugate

Photodynamic therapy (PDT) of cancer involves the systemic administration of a photoactive molecule known as a photosensitizer (PS), followed by illumination of the specified tissue volume with activating light of the correct wavelength to excite the PS, which in the presence of oxygen forms cytotoxic oxygen species that destroy the tumour (Hasan and Parrish, 1996). This tumour destruction can be due to direct cytotoxic effects on the tumour cells themselves, or to the destruction of the tumour blood supply due to constriction and stasis produced in the tumour vasculature depending primarily on whether the PS is located in the tumour interstitium or in the tumour vasculature (or for very early illumination times, in the blood) (Henderson and Dougherty, 1992). For commonly used PS, the relative amounts in the vasculature and the interstitium are governed by the time after administration at which illumination is performed (Boyle and Dolphin, 1996). These two mechanisms of tumour destruction can have different therapeutic effects on various sizes and types of tumours (Henderson et al, 1997). In addition to cancer, there is a growing number of applications of PDT to diseases where neovasculature needs to be destroyed, e.g. age-related macular degeneration (Schmidt-Erfurth et al, 1994), psoriasis (Calzavara-Pinton et al, 1996) and angiomas (Orenstein et al, 1990). An understanding of the factors governing the partitioning of the PS between blood vessels and tissue will broaden the potential applications of PDT and allow the rational design of PDT protocols. The ideal method of gaining information on this partitioning behaviour would be non-destructive and provide kinetic data in real time.

*Received 12 November 1998*
*Revised 11 March 1999*
*Accepted 22 March 1999*

*Correspondence to:* T Hasan

Although the tetrapyrrole compounds commonly used as PS have some selectivity for tumours (Hamblin and Newman, 1994a), means are being sought to increase this selectivity by using conjugates between PS and macromolecular carriers with targeting properties (Hasan, 1992). These targeting species include monoclonal antibodies (Duska et al, 1997), lipoproteins (Hamblin and Newman, 1994*b*), microspheres (Bachor et al, 1991), liposomes (Cuomo et al, 1990) and polymers (Krinick et al, 1994). The latter may be either natural polymers such as dextran (Rakestraw et al, 1990) and polyamino-acids (Goff et al, 1991; Soukos et al, 1997) or synthetic polymers such as *N*-(2-hydroxypropyl)-methacrylamide (Seymour et al, 1987) and polyvinyl alcohol (Davis et al, 1993). Polymer–drug conjugates have been proposed as targeting vehicles for delivery of cytotoxic drugs to tumours (Kopecek, 1991; Duncan and Spreafico, 1994). Advantages claimed include decreased systemic toxicity (Zunino et al, 1987; Yeung et al, 1991), and selective accumulation in tumours due to the phenomenon known as enhanced permeability and retention (Dvorak, 1986; Seymour, 1992). The increased permeability of tumour blood vessels is also responsible for the *initial* selective tumour-localizing ability of many macromolecules, such as Evans blue-albumin (Matsumura and Maeda, 1986), monoclonal antibodies (Dvorak et al, 1991), lipoproteins (Leppala et al, 1995) and small liposomes (Yuan et al, 1994). Enhanced retention of polymeric drugs is thought primarily to be due to the absence of an adequate system of lymphatic vessels (which would remove non-bound macromolecules) in solid tumours (Butler et al, 1975). The selective retention of other targeting species such as drugs bound to monoclonal antibodies (Mariani et al, 1995) or lipoproteins (Masquelier et al, 1986) has an additional component due to

*Present address: Department of Urology, National Tokyo Medical Center, 2-5-1 Higashigaoka, Meguro Ccity, Tokyo 152-0021, Japan interactions of these proteins with specific antigens or receptors present on cells in the tumour.

The preparation of conjugates between tetrapyrrole PS and polymers poses special challenges. They may aggregate to a greater or lesser extent due to the amphiphilic nature of hydrophobic tetrapyrroles joined to hydrophilic polymer chains. Water-soluble PS–polymer conjugates must be relatively hydrophilic, but may bear net cationic, anionic or neutral charges (Soukos et al, 1997). They may also be prepared with varying size distributions according to the molecular weight range of the original polymer. Varying the net charge and the molecular weight range of the conjugates may be expected to have profound effects on the kinetics of transport of PS out of the bloodstream and into the tumour. These questions are difficult to answer in vivo, and are the subject of the present study.

Because all PS are to a greater or lesser degree fluorescent, in vivo fluorescence microscopy is an attractive non-destructive method for obtaining pharmacokinetic information on the transport and distribution of PS in real time. This report describes the first use of in vivo laser scanning fluorescence microscopy to follow the transport of macromolecular–PS conjugates in vivo, and the first use in an orthotopic tumour model. These conjugates between chlorin *e6* (c*e6*) and poly-l-lysine (pl) had two sizes, being derived from pl chains of average $M_r$ 5000 and 25 000 and referred to hereafter as ‘small’ and ‘large’, and also had opposite charges, unmodified, referred to as ‘cationic’, and succinylated, referred to as ‘anionic’. An orthotopic rat prostate cancer model derived from MatLyLu cells (Vieweg et al, 1994) was selected because this model is metastatic in advanced stages, and allows the testing of treatments for effects on both local control and distant metastasis. In addition, the tumour was amenable to in vivo fluorescence microscopy presenting a 1–2 cm relatively flat-topped tumour for imaging. There is also evidence that orthotopic animal tumours may have vasculature that is more representative of that found in human tumours than subcutaneous implanted tumours (Fukumura et al, 1997).

### MATERIALS AND METHODS

### Cells

R-3327 MatLyLu (a cell line derived from the Dunning rat prostate cancer) was a generous gift from Dr WD Heston (Urologic Oncology Research Laboratory, Memorial Sloan-Kettering Cancer Center, New York, NY, USA). It was grown in RPMI-1640 with L-glutamine (Mediatech, Washington, DC, USA) supplemented with 10% fetal bovine serum (FCS) (Gibco, Grand Island, NY, USA), penicillin (100 units $ml^{-1}$) (Sigma, St Louis, MO, USA) and streptomycin (100 μg $ml^{-1}$) (Sigma). Cells were grown at 37°C in an atmosphere of 5% carbon dioxide and were passaged every 4–5 days. The continuous human endothelial hybrid cell line EA.hy926 (Edgell et al, 1983) was a generous gift of Dr C-JS Edgell (Department of Pathology, University of North Carolina, Chapel Hill, NC, USA). Cells were grown in Dulbecco’s modified Eagle’s medium (DMEM) with high glucose (Gibco) supplemented with heat-inactivated 10% FCS, penicillin, streptomycin and in the presence of HAT (100 mM hypoxanthine, 0.4 mM aminopterin, 16 mM thymidine (Gibco)). Medium was changed twice a week. Cells were subcultured weekly using trypsin–EDTA (Gibco) at a relative cell density of 1:16.

### Animal model

All animal procedures were approved by the Institutional Subcommittee on Research for Animal Care and were in compliance with the UKCCCR Guidelines (UKCCCR, 1998). There was no suffering or undue interference with the normal lifestyle of the rats. Experiments were carried out on male Copenhagen rats (2 months) (Harlan Sprague Dawley, Indianapolis, IN, USA). Rats were anaesthetized with ketamine cocktail (ketamine, xylazine and atropine) after slow induction with Metophane (methoxyflurane; Pitman-Moore, Washington Crossing, NJ, USA). After anaesthesia, a 2-cm longitudinal incision was made in a cranial direction starting from above the pubic bone, and the ventral lobe of the prostate was exposed by retracting the bladder. Then, $10^4$ MatLyLu cells in 0.1 ml of phosphate-buffered saline (PBS) were injected into the ventral lobe of the prostate. To prevent any spilled tumour cells from growing outside the prostate a few drops of povidone iodine solution 10% USP (Clinipad Corp., Guilford, CT, USA) was instilled in the injection site. The abdomen was closed with 2–0 silk. Three weeks after tumour cell injection almost spherical tumours had grown to 1–2 cm diameter (less than 5% body weight), completely replacing all normal prostate tissue in the ventral lobe, and substantial amounts of normal prostate in the dorsal lobe.

### Preparation and characterization of conjugates

The preparation of the conjugates has been described previously (Soukos et al, 1997). Briefly, poly-l-lysine HBr (polypeptide average $M_r$ 25 000, degree of polymerization (DP) = 196 or average $M_r$ 5000, DP = 40) was reacted with c*e6*-*N*-hydroxysuccinimide ester in dry dimethyl sulphoxide (DMSO) and either purified by extensive dialysis or further reacted with an excess of succinic anhydride before purification. The degree of c*e6* substitution on the polylysine chains was estimated by measuring the absorbance at 400 nm and calculating the amount of c*e6* present using $\varepsilon_{400\ nm}$ = 150 000 l $mol^{-1}$ $cm^{-1}$. The amount of polylysine was assumed to be the original quantity weighed out. After exhaustive dialysis it was assumed that the remaining c*e6* was covalently bound to the pl. Free c*e6* was dissolved in 0.1 M aqueous sodium hydroxide (NaOH), diluted in PBS and neutralized with 0.1 M hydrochloric acid.

### In vitro

MatLyLu or EA.hy926 cells were trypsinized in the exponential growth phase and counted using a haemocytometer. Then, $10^5$ cells in 1 ml growth medium with 10% FCS were seeded into each well of 24-well culture plates. These were returned to the incubator and kept at 37°C for 24 h to allow cells to attach and resume exponential growth, medium was removed and replaced with growth medium with 10% FCS containing conjugates or free c*e6* at a concentration of 0.4 μM c*e6* eq in triplicate wells, and again kept at 37°C. At all times the plates were protected from light by wrapping in aluminium foil. After 6 h had elapsed, the drug solution was aspirated from the wells, the cells were washed three times with 1 ml sterile PBS and incubated with 1 ml trypsin–EDTA for 10 min. The resulting cell suspension was then centrifuged, the trypsin supernatant was aspirated and retained, and the pellets (visibly fluorescent under long-wave UV) were dissolved in 1.5 ml 0.1 M NaOH per 1% sodium dodecyl sulphate

 © 1999 Cancer Research Campaign

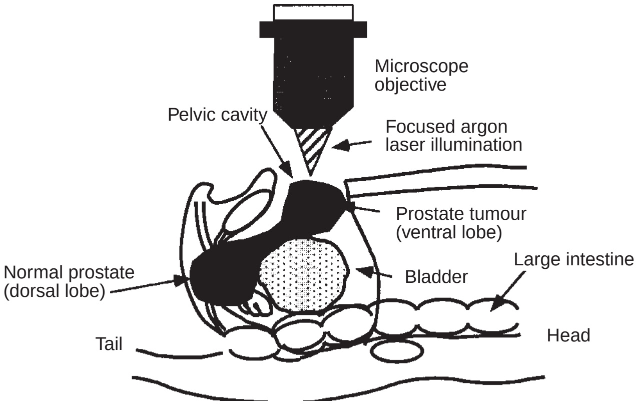

**Figure 1** Schematic of the in vivo imaging system for orthotopic rat prostate tumours

(SDS) for at least 24 h to give a homogenous solution. This treatment has been shown not to alter the fluorescence of the conjugates. The fluorescence of the cell extract was measured on a spectrofluorimeter (model FluoroMax, SPEX Industries, Edison, NJ, USA) and all measurements were made at concentrations at which the fluorescence versus concentration curve was linear. The excitation wavelength was 400 nm and the emission spectra of the cell suspensions were recorded from 580 to 700 nm. The fluorescence values were converted into mol equivalents of c*e6* by comparison with a calibration curve constructed with known amounts of each of the conjugates. The protein content of the entire cell extract was then determined by a modified Lowry (Larson et al, 1986) method using bovine serum albumin dissolved in 0.1 M NaOH per 1% SDS to construct calibration curves. The trypsin supernatant was also checked for the presence of fluorescence, which was negligible. Results were expressed as nmol of c*e6* per mg cell protein.

### Aggregation studies in serum

Sufficient conjugate was added to 2 aliquots of 1.5 ml pure horse serum (Gibco) to give a c*e6* eq concentration of 20 μM. Then, 750 μl of each aliquot were then serially diluted with 750 μl of horse serum 11 times to give two identical series of twofold serial dilutions from 20 μM to 10 nM. One set of dilutions was then centrifuged at 16 000 ***g*** for 15 min at 4°C while the other set was gently agitated. Three aliquots were taken from the supernatant of the centrifuged tubes and from the agitated tubes and each aliquot added to 1.4 ml 0.1 M NaOH per 1% SDS and the fluorescence measured as previously described. The fraction aggregated was calculated from the difference between the fluorescence in the supernatant of the centrifuged samples, and that in the agitated samples.

### Laser scanning fluorescence microscope

The laboratory prototype scanning laser microscope (SLM) was developed for video-rate (30 frames per second) fluorescence imaging of tumours and vasculature in vivo. The SLM is a modified version of a video-rate confocal scanning laser microscope that was originally built for reflectance imaging of tissue in vivo. By using a low numerical aperture objective lens and large detector aperture, we could image in a non-confocal mode.

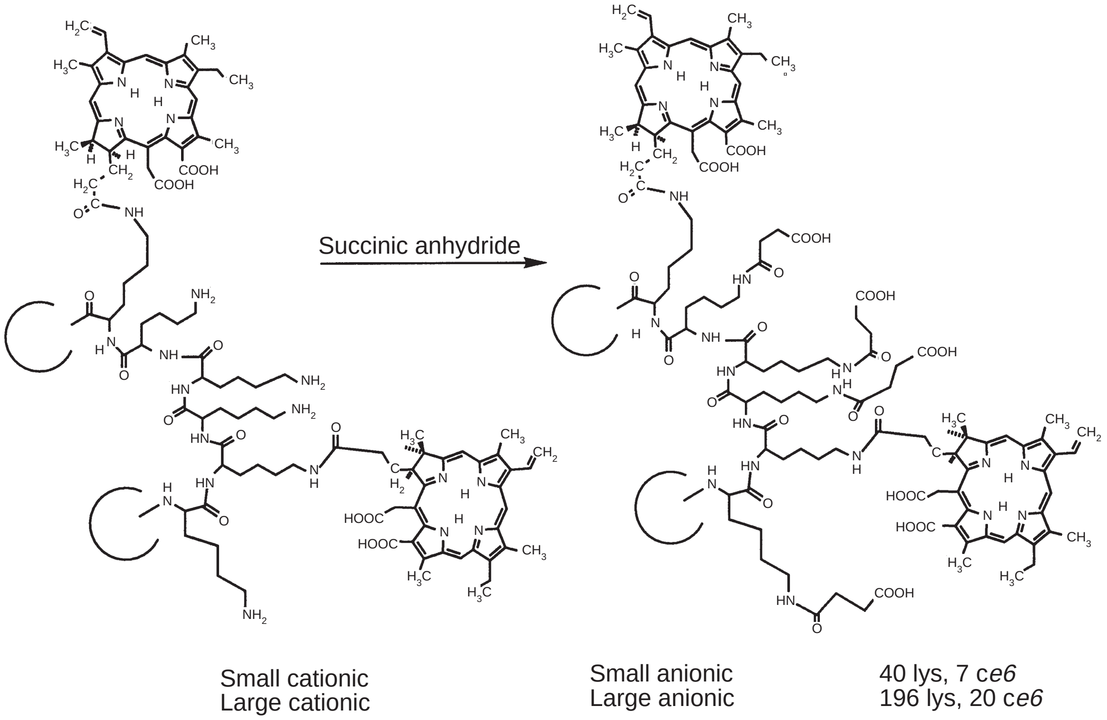

**Figure 2** Structure of the cationic and anionic conjugates

**Table 1** In vitro uptakes of the conjugates by prostate cancer cells and endothelial cells

| Conjugate | MatLyLu cells | Ea.hy926 cells |
|---|---|---|
| Free c*e6* | 0.005 ± 0.001 | 0.0015 ± 0.0003 |
| Large anionic | 0.2 ± 0.045 | 0.056 ± 0.004 |
| Small anionic | 0.26 ± 0.02 | 0.11 ± 0.022 |
| Large cationic | 1.93 ± 0.327 | 1.15 ± 0.216 |
| Small cationic | 3.3 ± 0.31 | 2.2 ± 0.314 |
| Large (ratio: cationic/anionic) | 9.65 ± 1.95 | 20.53 ± 4.25 |
| Small (ratio: cationic/anionic) | 12.7 ± 2.3 | 20.27 ± 3.7 |

Cells were incubated with conjugates at 1 μM c*e6* eq concentration in serum containing medium for 6 h at 37°C. Trypsinized cells were extracted and c*e6* quantified by fluorescence spectroscopy and cell protein measured by a modified Lowry procedure. Results are expressed as nmol c*e6* eq per mg cell protein. Values are means of two experiments each containing 3 wells and errors are s.e.m.

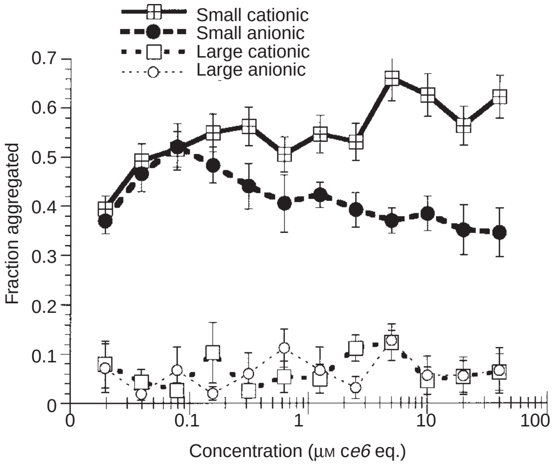

**Figure 3** Aggregation of the conjugates in serum. Values show the fraction of c*e6* eq which was pelleted after centrifugation at 16 000 ***g*** for 15 min at 4°C as a function of c*e6* concentration in 100% horse serum. The fraction aggregated was calculated from the difference between the fluorescence in the supernatant of the centrifuged and that in the agitated samples

Details of the optical design have previously been published (Rajadhyaksha et al, 1995). Illumination was obtained by coupling an argon-ion laser (Innova 100, Coherent, Palo Alto, CA, USA), into the SLM. Video-rate horizontal and vertical scanning was with a rotating polygon mirror (model M225030XLIM with controller VFC2, Lincoln Laser, Phoenix, AZ, USA) and an oscillating galvanometric mirror (model G325DT, General Scanning, Watertown, MA, USA) respectively. The intermediate optics in the scan system consisted of folding mirrors and achromatic lenses. The detector was a silicon avalanche photodiode (model C39050E, EG&G Optoelectronics, Quebec, Canada). The detector output was sent to a video monitor and video storage devices such as a S-VHS videotape recorder (Panasonic AG-7300) and an eight bits per pixel frame grabber (model Pixelpipeline PTP425, Perceptics, Knoxville, TN, USA).

We used a 2.5×, 0.07 numerical aperture objective and a detector aperture of 1 mm diameter (i.e. 600 μm at the tissue.) The imaging was with 30 mW of argon-ion 454–528 nm multilines, and with a 570 nm long pass filter which transmitted only the fluorescent image to the detector. The images were ‘grabbed’ with the frame grabber, each grabbed image being an integration of four frames. Four frames is the optimum compromise between increasing the image signal-to-noise ratio and introducing blurring caused by animal motion. The frame-grabber gain was typically 200 and offset was 100 for grabbing the images. With the 2.5×, 0.07 numerical aperture lens, we measured a spot of diameter of 10 μm at the tissue. When the laser beam scans the field of view, each spot on the tissue is illuminated with 30 mW for approximately 400 ns. Thus each spot receives 0.02 J $cm^{-2}$ of energy, and grabbing each image required approximately 5 s. If we assume continuous illumination during this time over the field of view of 6.4 × 3.4 mm, the energy deposited on the tissue is 0.7 J $cm^{-2}$ for each image.

### In vivo fluorescence microscopy

Rats were anaesthetized as described above, and a 3 cm longitudinal incision was made in a cranial direction from the top of the *symphysis pubica*, which was sufficient to completely expose the tumour-bearing prostate. The skin of the abdominal incision was sutured with 1–0 silk to the skin of the inside of the thigh in order to permanently retract the incision for the duration of the experiment. A 5 cm square of surgical gauze was soaked in saline, rolled up and inserted into the upper abdomen to retain the intestines in place out of the observation field. Figure 1 shows a schematic representation of the imaging setup.

The conjugates or free c*e6* (250 μg c*e6* eq per 250 g rat) were dissolved in 1 ml of PBS and slowly injected (over at least 5 min) via the penile vein. Images were captured as described above, and rats were kept warm in between imaging. When rats showed any sign of regaining consciousness they received additional anaesthesia via inhalational metophane. At the completion of the experiment, rats were sacrificed with a large overdose of intravenous ketamine. At least three rats were imaged for each of five compounds. Imaging usually continued for as long as the fluorescence levels in the images continued to increase. This time varied from 90 min to 8 h.

### Image processing

Images were captured with comparable levels of argon-laser power and gain settings so the grey scale levels in subsequent images were directly comparable. Since it was apparent that the levels of fluorescence observed in any single tumour were somewhat inhomogenous, at least two areas were chosen for imaging in each tumour. Due to the necessity of having a relatively level tumour surface for imaging it was seldom possible to have more than three observation fields in any single tumour without significant overlap. Images were divided into three compartments, blood vessel interior, blood vessel wall and tumour tissue. For each image the grey scale values of each compartment were measured by averaging the values of at least 100 pixels in each compartment including the most and least fluorescent pixels. Means and standard errors were then generated from the 100-pixel values from images from each of three rats at each time point.

### RESULTS

### Preparation of conjugates

The large polylysine chain (average $M_r$ 25 000, DP = 196), was found to have an average of 20 c*e6* molecules attached per chain

 © 1999 Cancer Research Campaign

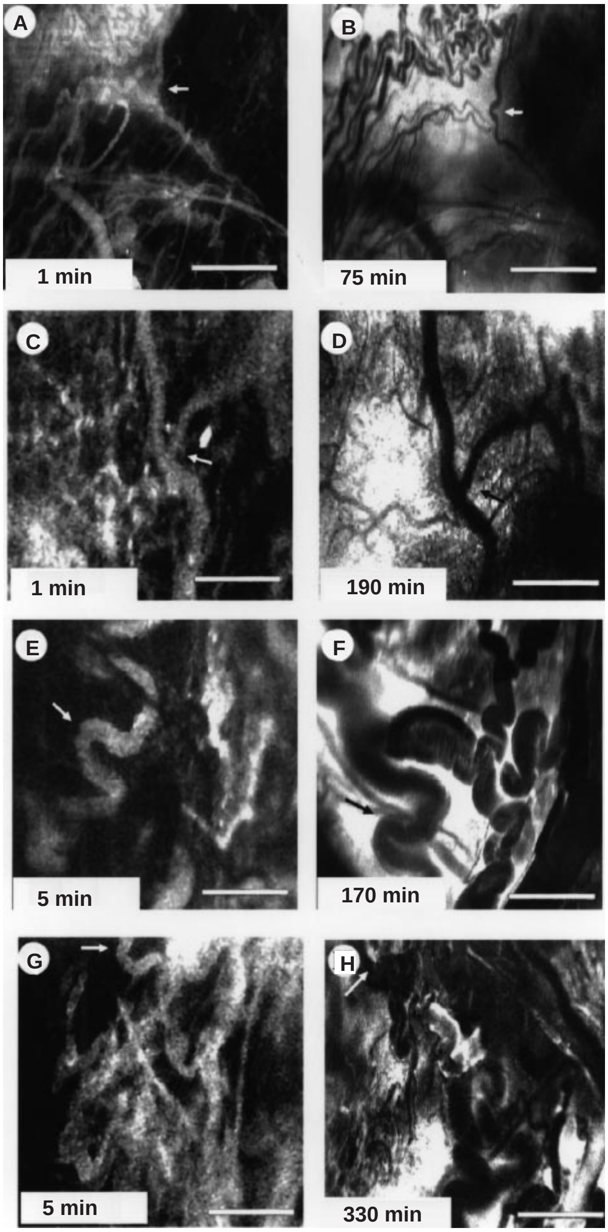

**Figure 4** Pairs of images from laser scanning fluorescence microscopy, one at an early time point and one at a late time point. Arrows indicate landmarks for orientation in pairs of images. The grey scale values of these images have been optimized for brightness and contrast for printing, and are only proportional to the grey scale values which were used for calculation of pharmacokinetics. (**A, B**) Free c*e6*; (**C, D**) Small anionic; (**E, F**) Large cationic; (**G, H**) Small cationic. Scale bar is 500 μm in every case thus raising the average $M_r$ to approximately 37 000 for the large cationic species, and after succinylation to approximately 54 500 for the large anionic species assuming complete succinylation. The small polylysine chain (average $M_r$ 5000, DP = 40) had an average of 7 c*e6* molecules attached per chain, thus giving $M_r$ values of approximately 9000 and 12 000 for the small cationic and small anionic species respectively. The structures are shown in Figure 2.

### In vitro uptake of conjugates

Uptake experiments were performed to investigate if there were any differences in the interaction of the conjugates with endothelial cells and prostate cancer cells which could account for observed differences in the pharmacokinetics in vivo. The results (Table 1) showed that the uptake of *ce6* by both cell lines was many times higher for the cationic conjugates than the anionic conjugates, which in turn had much higher uptake than free *ce6*. Both cell lines took up significantly more c*e6* from the small cationic conjugate than the large, while the difference in uptake between the large and small anionic conjugate was less pronounced. The prostate cancer cells in every case took up more c*e6* than the endothelial cells, but when the ratio of uptake from the cationic to anionic conjugates was examined (Table 1) it can be seen that in fact the endothelial cells had a greater preference for the cationic conjugate of both sizes than the prostate cancer cells.

### Aggregation in serum

In order to gain some insight into the behaviour of these conjugates in the bloodstream, we carried out a series of experiments to investigate their aggregation behaviour in 100% horse serum. All the conjugates are highly aggregated in PBS but it was visually apparent that there were differences between the conjugates in serum. Since the aggregates formed by the small conjugates were sufficiently large to fall out of solution, we compared their behaviour after centrifugation with that after gentle agitation. The results are shown in Figure 3. It can be seen that there is a very significant difference between the large and small conjugates of both charges. The large conjugates are almost completely disaggregated in serum, while the small conjugates are between 40 and 60% aggregated. There is a difference in the extent of aggregation between the small cationic and small anionic conjugates at the higher concentrations. It should be noted that assuming the plasma volume of a 250 g rat is 10 ml, then the initial concentration of c*e6* eq after injection of 1 mg $kg^{-1}$ body weight is approximately 40 μM.

### Intratumoural transport and pharmacokinetics by fluorescence imaging

Examples of images captured from the fluorescence imaging studies are shown in Figure 4 A–H. The initial images (Figure 4 A,C,E,G) were usually less fluorescent but in better focus than the intermediate images, while the focus improved towards the end of the imaging (Figure 4 B,D,F,H) when the vasculature became negative with respect to the rest of the tissue. Considerable heterogeneity was observed in the degree to which different types of tumour vasculature extravasated fluorescence. Areas where the blood vessels were convoluted were especially ‘leaky’ (Figure 4 A,B), while large straight vessels were less leaky and small straight vessels the least. Fluorescence can be seen on the vessel wall in Figure 4 E,G. With some conjugates there was a significant ‘lag’ period after the fluorescence had leaked out into the tissue, and the blood vessels had become negative, before the fluorescence intensity in the tissue rose dramatically.

The fluorescence imaging technique allows the preparation of relative pharmacokinetic curves for three separate compartments of the tumour; the interior of the blood vessels, the walls of the blood vessels and the tumour interstitium. The curves for the increase in fluorescence in the tissue compartment from the four conjugates and free chlorin *e6* is shown in Figure 5. The fluorescence in the blood vessel interior reached a peak shortly after injection (2–10 min) and reduced fairly rapidly, dropping to a low value in every case by 50–100 min after injection. At some time

*© 1999 Cancer Research Campaign* 

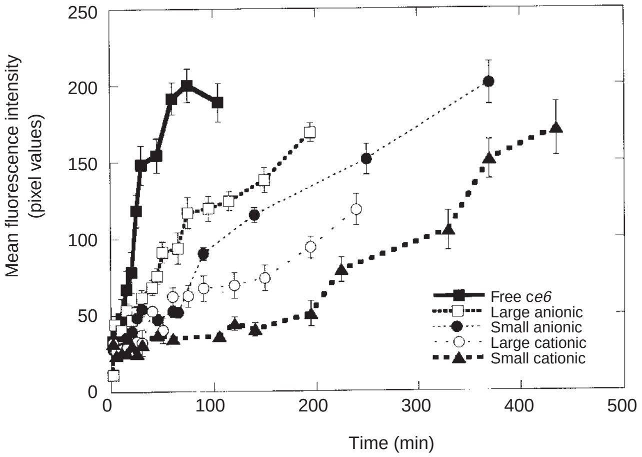

**Figure 5** Time-dependent increase of fluorescence in the tumour interstitium. The grey scale levels of at least 100 pixels in the tissue compartment were averaged for each image, values are the means of these mean values for three rats at each time point, and bars are s.e.m.

after injection the fluorescence could be observed to localize on the walls of the blood vessels, and the peak of this localization occurred between 20 and 40 min after injection, depending on the conjugate injected. The peak intensity of the vessel wall fluorescence was always higher than the peak observed in the interior of the blood vessels. The vessel wall fluorescence remained strong only for a period of 30–50 min before disappearing. The fluorescence in the tissue between the blood vessels increased at distinct rates for each injected species (Figure 5). The relative rates of increase of tissue fluorescence could be illustrated by the times required for the fluorescence to reach a mean graey scale level of 100 in the tissue and were as follows: free c*e6* (20 min) > large anionic (63 min) > small anionic (107 min) > large cationic (202 min) > small cationic (307 min).

### DISCUSSION

The rate and extent to which intravenously injected PS partition from the vasculature into tumour tissue can have important implications for the optimum time for light delivery in PDT. This study has shown that there are dramatic and surprising effects of both size and charge of the polymer–PS conjugates on the pharmacokinetics of fluorescence distribution between vasculature and interstitium in an orthotopic rat prostate tumour model. This was feasible because in vivo laser scanning fluorescence microscopy provides a non-destructive method of gaining pharmacokinetic information in real time.

In vitro studies showed that, although the prostate tumour cells took up more of the conjugates of both sizes and charges than the endothelial cells, in fact the ratios of the uptakes of the cationic to anionic was higher for the endothelial cells than the prostate cancer cells. This implies that, although both cell types are negatively charged, the endothelial cells have a relatively higher negative charge than the prostate cells, while the latter have a greater capacity for endocytosis. This higher binding of the cationic conjugates to endothelial cells is in agreement with literature reports that endothelial cells express anionic regions on the glycocalyx (Haldenby et al, 1994), fenestral membranes (Ghinea and Simionescu, 1985) and vesicular openings (Baldwin and Chien, 1984), all of which have a pronounced tendency to bind polycations. These anionic regions are thought to play a role in modifying permeability and transcytosis of macromolecules (Simionescu and Simionescu, 1986). The reason for the differences in cellular uptake between the small and large conjugates of the same charge was less clear. The most likely explanation is provided by the observed difference in the degree of aggregation between the small and large conjugates of both charges. The aggregation behaviour of these conjugates is complicated and highly dependent on solvent (MR Hamblin and T Hasan, unpublished results); however, in 100% serum the small conjugates are significantly more aggregated than the larger ones (Figure 4), which may explain their higher cellular uptake. Aggregates may bind strongly to the plasma membrane or be internalized by phagocytosis. An alternative explanation is that the increased uptake of the small cationic compared to the large cationic is due to more pronounced endocytosis of smaller molecules. The extremely low cellular uptake of free c*e6* compared to that observed from the conjugates is in agreement with previous reports with similar conjugates (Soukos et al, 1997). Considering these in vitro results, we predicted that the cationic conjugates when injected in vivo, should bind to the endothelial cells in the tumour blood vessels, while the anionic conjugates should bind less to the endothelium, and therefore should be able to pass through the walls of the vessel faster. We supposed that the small conjugates would extravasate faster than the large ones of the same charge, as suggested by several previous studies on the molecular size dependence of extravasation (Mayhan and Heistad, 1985; Ley and Arfors, 1986; Yuan et al, 1995).

There are differences of opinion as to the extent to which in vivo measurements of fluorescence can be correlated with the concentration of fluorophore in tissue (Bernstein et al, 1991; Frisoli et al, 1993), and to what extent it can be correlated with photodynamic efficacy (van Leengoed et al, 1994). The attenuation of fluorescence in tissue can be due to absorption and/or scattering of both excitation and emission light. It is clear that this applies to some extent to the blood vessels in the present study. At early time points, when the majority of fluorescence is still in the vessel interior or vessel wall, the total intensity of fluorescence is significantly less than at later time points. The marked increase of tissue fluorescence occurring some time after the fluorescence appeared to have ceased moving through the blood vessel walls may be due either to an increase in the fluorescence quantum yield, or to a lessening in the extent to which the fluorescence is reduced due to absorption and scattering. In the former case, the fluorescence quantum yield could increase because the polymeric conjugates have been metabolized by, for example, lysosomal proteases (Krinick et al, 1994), to free c*e6* which has a greater fluorescence quantum yield (data not shown), or because the conjugates underwent a disaggregation process, similar to that which has been reported to occur with Photofrin® (Bottiroli et al, 1990). In the latter case, the increased distance of the fluorophore from the blood vessels could have reduced the scattering of excitation or emission light by erythrocytes, or absorption of excitation or emission light by haemoglobin.

Although there is broad agreement on the fact that certain types of tumour vasculature have dramatically increased permeability to macromolecules (Dvorak et al, 1988; Yuan et al, 1994), and that this is the result of tumour-secreted angiogenesis factors such as vascular endothelial growth factor (Senger et al, 1993), the precise mechanism of the hyperpermeability is not certain (Hobbs et al,

© 1999 Cancer Research Campaign

1998). Transport of molecules out of tumour blood vessels can be a combination of a paracellular process (Jain, 1987), when it is due to discontinuous endothelium (Jain, 1988), wide inter-endothelial cell junctions (Jain, 1989) and fenestrations (Roberts and Palade, 1995), and a transcellular process (Kohn et al, 1992) when it is due to increased expression and function of a system of vesiculo-vacuolar organelles which transport macromolecules from the apical to the basal membrane (Dvorak et al, 1996). The mechanism operating may have implications on the charge selectivity of transendothelial transport. It has been shown that membrane of the vesicular–vacuolar neck has no strongly anionic residues (Simionescu et al, 1981), thus allowing negatively charged macromolecules to penetrate into the vesicle, while cationic macromolecules remain bound to other regions of the luminal endothelial surface (Ghinea and Simionescu, 1985). This would explain the faster extravasation of the anionic conjugates compared to the cationic ones.

All reports (Ley and Arfors, 1986) that have investigated the size dependence of the rate of extravasation of macromolecules both in tumour (Yuan et al, 1995) and normal vasculature (Mayhan and Heistad, 1985), have concluded that small molecules come out of the blood vessels faster than large ones, and the Stokes–Einstein radius is thought to be a good predictor of extravasation of neutral macromolecules (Nugent and Jain, 1984; Ojteg et al, 1987). (However, there have been no reports on the size dependence of rates of extravasation of molecules that contain tetrapyrrole structures with their innate tendency to aggregate.) Considering these reports we were very surprised to find that the large conjugates of both charges gave faster fluorescence increase than the equivalently charged small conjugates. One explanation of this observation is that the small conjugates in fact behave as if they were larger than the large ones, and corroboration of this hypothesis is gained from the aggregation experiments in 100% serum, where the large conjugates were completely disaggregated, while the small ones remained more than 40–60% aggregated. Presumably this disaggregation is an equilibrium process as, in vivo, all the fluorescence eventually came out of the blood vessels, the small conjugates just taking considerably longer than their large analogues. Other explanations could include differences in spatial conformations and modes of folding of the larger conjugates compared to the smaller, which could lead to shielding of charges or alteration of hydrophobic interactions.

In summary, we have shown that the use of the in vivo laser scanning fluorescence microscope can give real-time information on the rate of extravasation of PS and PS conjugates. The knowledge of the rate of extravasation of PS of varying structures, and their distribution between the vascular and parenchymal compartments of the tumour could have implications for the timing of light delivery in PDT. Previous reports have deduced this rate and partitioning indirectly by measuring serum half-lives (Bellnier et al, 1993) and by characterizing photodynamic tissue damage as predominantly mediated by vascular effects or by tumour cell damage (Henderson et al, 1997). Applications utilizing the PDT-mediated destruction of unwanted neo-vasculature, such as in age-related macular degeneration (Schmidt-Erfurth et al, 1994), require that the PS be localized in or near the blood vessels. Conversely, it has been suggested that to gain the best tumouricidal effect from PDT it is necessary to have a combination of vascular effects and direct tumour cell killing effects (Henderson et al, 1997). The next step in this study is to measure actual tissue concentrations of c*e6* and then to treat tumours in this animal model with these conjugates, and correlate the physiological response with the time of light delivery and the precise structure of the conjugate injected.

### ACKNOWLEDGEMENTS

This work was supported by a grant from the NIH-R01 AR40352, DoD MFEL contract number N 00014-94-1-0927, DOE contract number DE-FG02-91-ER61228, and the MGH Laser Center (partial fellowship to TM). We are grateful to Brian W. Pogue for help and advice and to Philip S Newsome and Pradeep Penta for technical assistance. We thank Dr WD Heston and Dr C-JS Edgell for the gift of cell lines, and Coherent Inc. for the loan of the argon-ion laser.

### REFERENCES

Bachor R, Shea CR, Gillies R and Hasan T (1991) Photosensitized destruction of human bladder carcinoma cells treated with chlorin e6-conjugated microspheres. *Proc Natl Acad Sci USA* **88**: 1580–1584

Baldwin AL and Chien S (1984) Endothelial transport of anionized and cationized ferritin in the rabbit thoracic aorta and vasa vasorum. *Arteriosclerosis* **4**: 372–382

Bellnier DA, Henderson BW, Pandey RK, Potter WR and Dougherty TJ (1993) Murine pharmacokinetics and antitumor efficacy of the photodynamic sensitizer 2-[1-hexyloxyethyl]-2-devinyl pyropheophorbide-a. *J Photochem Photobiol B* **20**: 55–61

Bernstein EF, Friauf WS, Smith PD, Cole JW, Solomon RE, Fessler JF, Thomas GF, Black C and Russo A (1991) Transcutaneous determination of tissue dihematoporphyrin ether content. A device to optimize photodynamic therapy. *Arch Dermatol* **127**: 1794–1798

Bottiroli G, Croce AC and Vaghi P (1990) Equilibrium among hematoporphyrin derivative components. II. Effect of esterase activity. *Photochem Photobiol* **51**: 169–174

Boyle RW and Dolphin D (1996) Structure and biodistribution relationships of photodynamic sensitizers. *Photochem Photobiol* **64**: 469–485

Butler TP, Grantham FH and Gullino PM (1975) Bulk transfer of fluid in the interstitial compartment of mammary tumors. *Cancer Res* **35**: 3084–3088

Calzavara-Pinton PG, Szeimies RM, Ortel B and Zane C (1996) Photodynamic therapy with systemic administration of photosensitizers in dermatology. *J Photochem Photobiol B*, **36**: 225–231

Cuomo V, Jori G, Rihter B, Kenney ME and Rodgers MA (1990) Liposome-delivered Si(IV)-naphthalocyanine as a photodynamic sensitiser for experimental tumours: pharmacokinetic and phototherapeutic studies. *Br J Cancer* **62**: 966–970

Davis N, Liu D, Jain AK, Jiang SY, Jiang F, Richter A and Levy JG (1993) Modified polyvinyl alcohol-benzoporphyrin derivative conjugates as phototoxic agents. *Photochem Photobiol* **57**: 641–647

Duncan R and Spreafico F (1994) Polymer conjugates. Pharmacokinetic considerations for design and development. *Clin Pharmacokinet* **27**: 290–306

Duska LR, Hamblin MR, Bamberg MP and Hasan T (1997) Biodistribution of charged F(ab′)2 photoimmunoconjugates in a xenograft model of ovarian cancer. *Br J Cancer* **75**: 837–844

Dvorak AM, Kohn S, Morgan ES, Fox P, Nagy JA and Dvorak HF (1996) The vesiculo-vacuolar organelle (VVO): a distinct endothelial cell structure that provides a transcellular pathway for macromolecular extravasation. *J Leukoc Biol* **59**: 100–115

Dvorak HF (1986) Tumors: wounds that do not heal. Similarities between tumor stroma generation and wound healing. *N Engl J Med* **315**: 1650–1659

Dvorak HF, Nagy JA and Dvorak AM (1991) Structure of solid tumors and their vasculature: implications for therapy with monoclonal antibodies. *Cancer Cells* **3**: 77–85

Dvorak HF, Nagy JA, Dvorak JT and Dvorak AM (1988) Identification and characterization of the blood vessels of solid tumors that are leaky to circulating macromolecules. *Am J Pathol* **133**: 95–109

Edgell CJ, Mcdonald CC and Graham JB (1983) Permanent cell line expressing human factor VIII-related antigen established by hybridization. *Proc Natl Acad Sci USA* **80**: 3734–3737

© 1999 Cancer Research Campaign 

Frisoli JK, Tudor EG, Flotte TJ, Hasan T, Deutsch TF and Schomacker KT (1993) Pharmacokinetics of a fluorescent drug using laser-induced fluorescence. *Cancer Res* **53**: 5954–5961

Fukumura D, Yuan F, Monsky WL, Chen Y and Jain RK (1997) Effect of host microenvironment on the microcirculation of human colon adenocarcinoma. *Am J Pathol* **151**: 679–688

Ghinea N and Simionescu N (1985) Anionized and cationized heme undecapeptides as probes for cell surface charge and permeability studies: differentiated labeling of endothelial plasmalemmal vesicles. *J Cell Biol* **100**: 606–612

Goff BA, Bamberg M and Hasan T (1991) Photoimmunotherapy of human ovarian carcinoma cells ex vivo. *Cancer Res* **51**: 4762–477

Haldenby KA, Chappell DC, Winlove CP, Parker KH and Firth JA (1994) Focal and regional variations in the composition of the glycocalyx of large vessel endothelium. *J Vasc Res* **31**: 2–9

Hamblin MR and Newman EL (1994*a*) On the mechanism of the tumour-localising effect in photodynamic therapy. *J Photochem Photobiol B* **23**: 3–8

Hamblin MR and Newman EL (1994*b*) Photosensitizer targeting in photodynamic therapy. II. Conjugates of haematoporphyrin with serum lipoproteins *J Photochem Photobiol B* **26**: 147–157

Hasan T (1992) Photosensitizer delivery mediated by macromolecular carrier systems. In: *Photodynamic Therapy: Basic Principles and Clinical Applications*, Henderson B and Dougherty T (eds), pp. 187–200. Marcel Dekker

Hasan T and Parrish JA (1996) Photodynamic therapy of cancer. In: *Cancer Medicine*, Holland JF, Frei EI, Bast RCJ, Kufe DW, Morton DL and Weichselbaum RR (eds), pp. 739–751. Williams & Wilkins: Baltimore, MD

Henderson BW and Dougherty TJ (1992) How does photodynamic therapy work? *Photochem Photobiol* **55**: 145–157

Henderson BW, Bellnier DA, Greco WR, Sharma A, Pandey RK, Vaughan LA, Weishaupt KR and Dougherty TJ (1997) An in vivo quantitative structure–activity relationship for a congeneric series of pyropheophorbide derivatives as photosensitizers for photodynamic therapy. *Cancer Res* **57**: 4000–4007

Hobbs SK, Monsky WL, Yuan F, Roberts WG, Griffith L, Torchilin VP and Jain R (1998) Regulation of transport pathways in tumor vessels: role of tumor type and microenvironment. *Proc Natl Acad Sci USA* **95**: 4607–4612

Jain RK (1987) Transport of molecules across tumor vasculature. *Cancer Metastasis Rev* **6**: 559–593

Jain RK (1988) Determinants of tumor blood flow: a review. *Cancer Res* **48**: 2641–2658

Jain RK (1989) Delivery of novel therapeutic agents in tumors: physiological barriers and strategies. *J Natl Cancer Inst* **81**: 570–576

Kohn S, Nagy JA, Dvorak HF and Dvorak AM (1992) Pathways of macromolecular tracer transport across venules and small veins. Structural basis for the hyperpermeability of tumor blood vessels. *Lab Invest* **67**: 596–607

Kopecek J (1991) Targetable polymeric anticancer drugs. Temporal control of drug activity. *Ann N Y Acad Sci* **618**: 335–344

Krinick NL, Sun Y, Joyner D, Spikes JD, Straight RC and Kopecek J (1994) A polymeric drug delivery system for the simultaneous delivery of drugs activatable by enzymes and/or light. *J Biomater Sci Polym Ed* **5**: 303–324

Larson E, Howlett B and Jagendorf A (1986) Artificial reductant enhancement of the Lowry method for protein determination. *Anal Biochem* **155**: 243–248

Leppala J, Kallio M, Nikula T, Nikkinen P, Liewendahl K, Jaaskelainen J, Savolainen S, Gylling H, Hiltunen J, Callaway J, Kahl S and Farkkila M (1995) Accumulation of 99mTc-low-density lipoprotein in human malignant glioma. *Br J Cancer* **71**: 383–387

Ley K and Arfors KE (1986) Segmental differences of microvascular permeability for FITC-dextrans measured in the hamster cheek pouch. *Microvasc Res* **31**: 84–99

Mariani G, Molea N, Bacciardi D, Boggi U, Fornaciari G, Campani D, Salvadori PA, Giulianotti PC, Mosca F, Gold DV, Sharkey RM and Goldenberg DM (1995) Initial tumor targeting, biodistribution, and pharmacokinetic evaluation of the monoclonal antibody PAM4 in patients with pancreatic cancer. *Cancer Res* **55**: 5911s–5915s

Masquelier M, Vitols S and Peterson C (1986) Low-density lipoprotein as a carrier of antitumoral drugs: in vivo fate of drug-human low-density lipoprotein complexes in mice. *Cancer Res* **46**: 3842–3847

Matsumura Y and Maeda H (1986) A new concept for macromolecular therapeutics in cancer chemotherapy: mechanism of tumoritropic accumulation of proteins and the antitumor agent smancs. *Cancer Res* **46**: 6387–6392

Mayhan WG and Heistad DD (1985) Permeability of blood–brain barrier to various sized molecules. *Am J Physiol* **248**: H712–718

Nugent LJ and Jain RK (1984) Plasma pharmacokinetics and interstitial diffusion of macromolecules in a capillary bed. *Am J Physiol* **246**: H129–137

Ojteg G, Nygren K and Wolgast M (1987) Permeability of renal capillaries. II. Transport of neutral and charged protein molecular probes. *Acta Physiol Scand* **129**: 287–294

Orenstein A, Nelson JS, Liaw LH, Kaplan R, Kimel S and Berns MW (1990) Photochemotherapy of hypervascular dermal lesions: a possible alternative to photothermal therapy? *Lasers Surg Med* **10**: 334–343

Rajadhyaksha M, Grossman M, Esterowitz D, Webb RH and Anderson RR (1995) In vivo confocal scanning laser microscopy of human skin: melanin provides strong contrast. *J Invest Dermatol* **104**: 946–952

Rakestraw SL, Tompkins RG and Yarmush ML (1990) Preparation and characterization of immunoconjugates for antibody-targeted photolysis. *Bioconjug Chem* **1**: 212–221

Roberts WG and Palade GE (1995) Increased microvascular permeability and endothelial fenestration induced by vascular endothelial growth factor. *J Cell Sci* **108**: 2369–2379

Schmidt-Erfurth U, Hasan T, Gragoudas E, Michaud N, Flotte TJ and Birngruber R (1994) Vascular targeting in photodynamic occlusion of subretinal vessels. *Ophthalmology* **101**: 1953–1961

Senger DR, Van de Water L, Brown LF, Nagy JA, Yeo KT, Yeo TK, Berse B, Jackman RW, Dvorak AM and Dvorak HF (1993) Vascular permeability factor (VPF, VEGF) in tumor biology. *Cancer Metastasis Rev* **12**: 303–324

Seymour LW (1992) Passive tumor targeting of soluble macromolecules and drug conjugates. *Crit Rev Ther Drug Carrier Syst* **9**: 135–187

Seymour LW, Duncan R, Kopeckova P and Kopecek J (1987) Daunomycin- and adriamycin-N-(2-hydroxypropyl)methacrylamide copolymer conjugates; toxicity reduction by improved drug-delivery. *Cancer Treat Rev* **14**: 319–327

Simionescu M and Simionescu N (1986) Functions of the endothelial cell surface. *Annu Rev Physiol* **48**: 279–293

Simionescu N, Simionescu M and Palade GE (1981) Differentiated microdomains on the luminal surface of the capillary endothelium. I. Preferential distribution of anionic sites. *J Cell Biol* **90**: 605–613

Soukos NS, Hamblin MR and Hasan T (1997) The effect of charge on cellular uptake and phototoxicity of polylysine chlorin(e6) conjugates. *Photochem Photobiol* **65**: 723–729

UKCCCR (1998) United Kingdom Co-ordinating Committee on Cancer Research (UKCCCR) guidelines for the welfare of animals in experimental neoplasia (second edition). *Br J Cancer* **77**: 1–10

van Leengoed HL, Cuomo V, Versteeg AA, van der Veen N, Jori G and Star WM (1994) In vivo fluorescence and photodynamic activity of zinc phthalocyanine administered in liposomes. *Br J Cancer* **69**: 840–845

Vieweg J, Heston WD, Gilboa E and Fair WR (1994) An experimental model simulating local recurrence and pelvic lymph node metastasis following orthotopic induction of prostate cancer. *Prostate* **24**: 291–298

Yeung TK, Hopewell JW, Simmonds RH, Seymour LW, Duncan R, Bellini O, Grandi M, Spreafico F, Strohalm J and Ulbrich K (1991) Reduced cardiotoxicity of doxorubicin given in the form of N-(2-hydroxypropyl) methacrylamide conjugates: an experimental study in the rat. *Cancer Chemother Pharmacol* **29**: 105–111

Yuan F, Leunig M, Huang SK, Berk DA, Papahadjopoulos D and Jain RK (1994) Microvascular permeability and interstitial penetration of sterically stabilized (stealth) liposomes in a human tumor xenograft. *Cancer Res* **54**: 3352–3356

Yuan F, Dellian M, Fukumura D, Leunig M, Berk DA, Torchilin VP and Jain RK (1995) Vascular permeability in a human tumor xenograft: molecular size dependence and cutoff size. *Cancer Res* **55**: 3752–3756

Zunino F, Pratesi G and Pezzoni G (1987) Increased therapeutic efficacy and reduced toxicity of doxorubicin linked to pyran copolymer via the side chain of the drug. *Cancer Treat Rep* **71**: 367–373

*© 1999 Cancer Research Campaign*
